# A retrospective radiographic and histopathologic study of pathology associated with impacted teeth and other regions in patients at the Department of Oral and Maxillofacial Surgery: An institutional study

**DOI:** 10.4317/jced.62028

**Published:** 2024-10-01

**Authors:** Jayant S. Landge, Pankajkumar R. Gavali, Kanchan M. Shah, Shelly Sharma

**Affiliations:** 1 Department of Oral and Maxillofacial surgery, Government dental college and hospital, Ghati medical campus, Aurangabad, India

## Abstract

**Background:**

To examine the prevalence and pattern of pathology related to impacted teeth and other areas in the Department of Oral and Maxillofacial Surgery of the Government Dental College in Chhatrapati Sambhajinagar.

**Material and Methods:**

This 5-year single-center retrospective cohort study was conducted in the department of oral and maxillofacial surgery at the government dental college and hospital in Chhatrapati Sambhajinagar between 2019 and 2024. Based on age, sex, area, presence of impacted tooth, radiological and histopathological report. Statistical analysis using the Statistical Package for the Social Sciences (SPSS) software (SPSS Inc., Chicago, IL, USA).

**Results:**

A study of 3469 patients’ panoramic radiographs revealed that 1696 patients had at least one impacted tooth. The third molar was the most common impacted tooth (89.4%), with a higher prevalence among women. Among the 28,72 impacted teeth, the dentigerous cyst (193) was the most common lesion. Histopathologically, out of 546 cases, infected dental cyst (23.6%) was the most common hard tissue lesion, while traumatic fibroma (6%) was the most common soft tissue lesion according to biopsy reports.

**Conclusions:**

A study using panoramic radiographs found that 48.8% of 3469 patients had impacted teeth. Only 36% of patients with impacted teeth reported symptoms, and 75% had problem on just one side. Biopsies in 546 patients revealed cysts and traumatic fibromas as common findings. The study suggests that biopsies are necessary to determine the type and severity of lesions for early treatment.

** Key words:**Radiographic analysis, Histopathological analysis, cyst, tumors, impaction.

## Introduction

In addition to restorative care, the most common oral surgical procedure in India is the extraction of impacted teeth. A tooth that was hindered from erupting in the proper position due to space restrictions, malposition, or other obstacles is termed an impacted tooth (1). Tooth impaction is a common occurrence, and its distribution and prevalence in the various jaw regions can vary greatly (2).

The prevalence of impacted teeth can be influenced by various factors, such as the specific age group being considered, the timing of dental eruption, and the radiographic criteria used to assess dental development and eruption. In a study conducted by Nordenram *et al*., most mandibular third molar removals were observed in patients in the age range of 20-29 years. When a tooth is impacted, it can cause a variety of complications including carious lesions, infection, damage to neighbouring teeth, periodontal disease, and potentially the formation of oral and maxillofacial cysts or tumors. (3). Cystic or neoplastic lesions have been discovered to develop near the affected tooth in 16% of cases, particularly during the second and third decades of life (4). Radiography continues to be the primary method used in the diagnosis of jaw lesions, with the panoramic view being the initial imaging technique used to assess impacted teeth and associated lesions (5) and also the histopathological study plays a crucial role in confirming a definitive clinical diagnosis, allowing appropriate treatment planning with accurate documentation. Clinical findings often resemble systemic disorders or early malignant lesions, which can lead to misdiagnosis as benign lesions and subsequent incorrect treatment planning. Therefore, relying on histopathological study is essential to ensure an accurate diagnosis and guide effective treatment strategies.

The purpose was to assess the pathology associated with impacted teeth and other regions in patients visiting the Department of Oral and Maxillofacial Surgery of the government dental college and hospital in Chhatrapati Sambhajinagar.

## Material and Methods

The study involved the retrieval of 3469 consecutive panoramic radiographs and 546 biopsy records from Indian patients who visited the reception and primary care clinic at the Govt Dental College and Hospital, Chha. Sambhajinagar, between 2019 and 2024. Patients aged 10 years and older were included in the study, as it is commonly believed that impacted teeth usually begin to emerge around this age. Referrals to oral maxillofacial surgery were made from external sources for significant pathologies or incidental findings related to the third molars or any impacted teeth and their associated pathologies.

All panoramic radiographs were captured using the CS 9300 CareStream machine. The radiographs were then examined to determine the number of impacted teeth and assess the conditions surrounding them. Teeth that were partially or completely covered by alveolar bone or had not erupted to a normal vertical position in the dental arch were classified as impacted. To measure the width of the follicles, a ruler was used, compensating for a radiograph magnification of 1.30. Follicles with a width less than 3 mm were considered within the normal range. Although it is possible to observe the soft tissue profile in relation to the third molar, there is currently a lack of standardized clinical criteria for evaluating the soft tissue associated with impacted teeth. Recognizing and addressing these difficulties in accurately documenting the clinical condition of soft tissue is crucial to supporting future studies. Only pathologies that were biopsied and identified as impacted teeth based on radiographic findings were included for the radiographic study.

In biopsy, a total of 546 biopsies, performed over a period of 5 years from 2019 to 2024, were recovered from the oral pathology department of our institute’s Department of Oral and Maxillofacial Surgery. These samples were immersed in 10% formalin solution and subsequently submitted to the laboratory. For each case, data including age, sex, clinical manifestations, location of the injury, and histopathological results were collected. In the exclusion, irrelevant diagnosis.

The collected data were entered into a spreadsheet (Excel 2000; Microsoft, USA) a descriptive statistical analysis was performed using Statistical Package for the Social Sciences (SPSS) software (SPSS Inc., Chicago, IL, USA), while ensuring the anonymity of patient identities. The study received approval from the institutional ethics committee.

## Results

Panoramic radiographs of 3469 patients aged 10 to 80 years who visited our institute (mean, 38.9 years) were examined. A total of 1696 (48.8%) patients (mean age 27 years) presented with at least one impacted tooth. The 20-to-29-year age group had the highest prevalence of tooth impaction (66.3%), but this decreased with increasing age. The male to female ratio of the study group was 1:2 (1158:2311), and the ratio for patients with impacted teeth was 1:1.14 (823:947). Of the 2872 impacted teeth, mandibular third molars were the most common (89.4%), followed by maxillary third molars (5.6%) and maxillary canines (3.3%). Thirty-four percent of the patients (206/608) presented clinical symptoms in their third molars; three quarters (154/206) had complaints relating to one tooth. only, and the rest had symptoms on both sides. The most frequent complaints were pain and swelling, which were found in 154 and 52 patients, respectively. Only 39 patients complained of food trapping, while 26 patients reported bleeding, ( Table 1, Table 2).

There were a total of 2872 impacted teeth, each tooth being part of one or more impacted teeth. The predominant pathology observed was dentigerous cyst (193), with a higher prevalence in the third molar mandibular area, followed by the maxillary canine region, typically affecting one side. Additionally, the odontogenic Keratocyst (22) exhibited one or more impacted teeth within the cystic lesion, while only three cases of unicystic ameloblastoma presented impacted teeth ( Table 3). Dentigerous cysts are observed on radiographic examination as unilateral radiolucent cysts of different sizes, with clearly defined sclerotic margins, and are typically related to the crown of an unerupted tooth. The radiographic patterns of dentigerous cysts in relation to the crown of the impacted tooth can be categorized into three types: central, lateral, and circumferential. Our research revealed that the central type was the most commonly encountered.

For biopsies a total of 546 histological diagnosis was made, with 234 (43%) derived from male patients and 311 (57%) from female patients with a ratio of 1: 1. 3. The series shows a slight female predominance. A noTable association was observed between age and type of lesion, and between age and anatomical location. The age range was kept from 10 to 80 years. The highest frequency of the disease was among the age group of 21 to 70years. Most of the lesions were found due to awareness of the presence of the lesion (70%), while the rest (30%) were found during clinical investigation. Most of the biopsies performed were excisional and nearly 40 different histological diagnoses were established ( Table 4).

The frequent hard tissue lesions were cystic in nature and, among these, the most common infected dental cyst (23.6%) followed by odontogenic keratocyst (9.1%) and dentigerous cyst (6.9 %). In addition, osteomyelitis (6.2 %), ameloblastoma (4.5 %) and pyogenic granuloma (2.5%).

In the soft tissue lesion, we recorded 33 (6 %) traumatic fibromas, then 20 (3.6%) mucocele the most prevalent malignant neoplasm occurring in the soft tissue was squamous cell carcinoma, which represents (1.6 %) of the total lesion. Other common soft tissue lesions found were hyperplasia (3.8%) and dysplasia (3.3%), respectively ( Table 5).

## Discussion

Dental panoramic tomography is used primarily to examine impacted teeth in patients with dental insurance from government hospitals due to cost (3,7,8). Another limitation related to the use of dental panoramic tomography to examine impacted teeth and related pathologies is the reliability of the evaluation when relying solely on the radiograph for diagnosis no CBCT was used. To maintain diagnostic precision, radiographic results were cross-checked with clinical records obtained through standard forms during regular examinations.

While this research may not be fully representative of the entire Indian population, the findings may still be valuable to healthcare providers working in primary health settings. The patients included in the study reflect the diverse range of dental cases seen in a dental hospital. The study revealed that 48.8% of the population had impacted teeth (3,9). The statistic presented in this study is relatively high compared to research that involved a wider age range of patients, which also includes individuals younger than 17 years. Clinical information was obtained solely from the dental teaching hospital in India, where dental panoramic tomography is a standard procedure for all new patients. This study differs from previous studies that focused solely on specific age categories (3,8,10-12). Patients of different age brackets were included in the study and the age distribution of the study group was consistent with that of the Indian population as a whole (CENSUS 2011).

This radiographic study revealed that more than 50% of the subjects were between 19 and 30 years old. This finding suggests a greater awareness of dental health among this particular group, likely due to the free dental care they received in government dental schools during their formative years. However, the large number of patients in their third decade may also haveled to a higher prevalence of impacted teeth in the study, (3,8-12). Among the impacted teeth, the third molar was the most common, followed by the upper canines and other types. The mandibular third molars comprised 89.4% of all impacted third molars. This study revealed that around 36% of patients with impacted teeth had symptoms, whereas Stanley *et al*. (13) found symptoms in only 8.4% of their patients. Three-quarters of the patients in this study experienced issues only on one side, with pain and swelling being the main complaints. The most common jaw lesion involving impacted teeth is dentigerous cysts, followed by odontogenic keratocysts. Unicystic ameloblastomas and radicular cysts are found less frequently with impacted teeth in our study. Among these lesions, the third mandibular molar is the most commonly affected tooth, with the maxillary canine the second most commonly affected tooth. Jaw lesions related to impacted teeth often present with a variety of radiographic features. Initially, these lesions are commonly observed as unilocular radiolucency, which can later develop into multilocular lesions with distinct borders.

In biopsies, among patients aged 21 to 50 years, 85.3% of lesions were found in this study. However, a study conducted by Satorres *et al*. (14) revealed that only 49% of the lesions were present among patients aged 40 to 60 years. In particular, there were significant differences in incidence between men (43%) and women (57%), with women showing a higher prevalence. Furthermore, our investigation revealed that infected dental cysts (23.6%) were the most reported cases, according to the department of pathology at our institute. Followed by odontogenic keratocysts (9.1%), which are the most prevalent type of odontogenic cyst compared to dentigerous cysts (6.9%). These conditions were predominantly found in individuals in their second and third decades of life, consistent with the findings of a study by De Souza *et al*. (15), except in our study, where the prevalence was higher among women. The dentigerous cyst, a type of developmental cyst that forms when the follicle surrounding an unerupted tooth expands, has a higher incidence in men. The mandibular posterior region and the anterior maxilla are the areas most frequently affected by this cyst. According to Jones *et al*. (19), the frequency of dentigerous cysts is often associated with impacted lower third molars and upper canines. In our study osteomyelitis (6.2 %), ameloblastoma (4.5 %) and pyogenic granuloma (2.5%) were found similarly, in a study by Satorres *et al*. (14), it was observed that of the 77 documented periapical alterations, 52% were classified as root cysts, while 48% were identified as periapical granulomas. The World Health Organization officially defined odontogenic keratocysts as intraosseous tumors of odontogenic origin in 2005 and introduced the term keratocystic odontogenic tumor for this specific type of cyst (16,17). This particular lesion represents 12% to 14% of all odontogenic cysts found in the jaws. Patients affected by this condition are generally around 42 years old, with the highest incidence observed in individuals in their second and fifth decades of life (16). Radiographically, keratocystic odontogenic tumors exhibit well-defined unilocular or multilocular radiolucencies with smooth or scalloped margins that are often corticated (16-18).

Our study revealed that traumatic fibromas were the predominant inflammatory or reactive lesion, representing 6% of cases, while mucoceles followed closely behind at 3.6%. Mucoceles were found most frequently on the lower lip and exhibited a higher prevalence in women compared to men. The etiology of these lesions includes local irritants, trauma, calculus, and hormonal imbalances, which contribute to their increased occurrence in women, especially during pregnancy (20).

## Conclusions

According to the radiographic study, the presence of impacted teeth was observed in 48.8% of the 3469 patients included in this study. The order in which the impacted tooth types were identified matched the findings of previous reports (3,9). However, there was a noTable inclination towards impacted mandibular third molars within the study population. Symptoms were only reported by 36% of patients with dental impaction, and 75% experienced problems limited to one side. The most frequently reported problems were pain and swelling. The most common discoveries in biopsies performed on 546 patients were cystic lesions and traumatic fibroma. It is essential to emphasize the importance of biopsies in the examination of different pathological factors, as they contribute significantly to the determination of the prognosis, the establishment of early diagnosis, and, ultimately, the implementation of effective treatment.

## Figures and Tables

**Table 1:** Prevalence of impacted teeth in different age groups.

Age	Total no. of patients	Impacted teeth
10-19	48	13 (28.3 %)
20-29	1870	1239 (66.3 %)
30-39	979	354 (36.2 %)
40-49	528	84 (16 %)
50-59	34	4 (12 %)
60-69	9	1 (11.1 %)
70-80	1	1 (0.1 %)
Total	3496	1696

**Table 2:** Distribution of the impacted tooth.

Tooth	Cases	Pathologies associated with impacted
Mandibular 3^rd^ molars	2570	Dentigerous cyst (160) Odontogenic Keratocyst (10) Radicular cyst (6) Ameloblastoma (2)
Maxillary 3^rd^ molars	146	Dentigerous cyst (3)
Maxillary canine	97	Dentigerous cyst (30) Odontogenic Keratocyst (5)
Mandibular premolars	6	Odontogenic Keratocyst (5) Ameloblastoma (1)
Maxillary premolar	3	-
Mandibular Canine	36	Odontogenic Keratocyst (2)
Mandibular first and second molar	3	-
Maxillary central and lateral incisors	6	-
Mandibular Central and lateral incisors	5	-
Maxillary first and second molar	0	-
Total	2872	-

**Table 3:** Pathologies associated with impacted teeth.

Pathologies associated with impacted	NO. of Cases
Dentigerous cyst	193
Odontogenic Keratocyst	22
Radicular Cyst	6
Ameloblastomas	3
Total	224

**Table 4:** Prevalence of biopsies in different age groups.

AGE	CASES
10-19	7
20-29	269
30-39	127
40-49	70
50-59	43
60-69	21
70-80	9

**Table 5:** Hard and soft tissue biopsies and their percentage.

Dignosis	No. Of Cases	Percentage
Adenoid cystic carcinoma	2	0.3
Ameloblastic fibroma	3	0.5
Ameloblastoma	25	4.5
Amelogenesis imperfecta	3	0.5
Central giant cell granuloma	28	5.1
Chronic nonspecific ulcer	5	0.9
Dentigerous cyst	38	6.9
Dysplasia	18	3.3
Hyperplasia	21	3.8
Infected dental cyst	129	23.6
Inflammatory hyperplasia	7	1.2
Intramucosal nevus	1	0.1
Leukoplakia	7	1.2
Lichen planus	3	0.5
Lichenoid reaction	3	0.5
Lipoma	3	0.5
Mucocele	20	3.6
Nasopalatine dental cyst	2	0.3
Odontogenic keratocyst	50	9.1
Odontogenic myxoma	5	0.9
Oral sub mucosal fibrosis	3	0.5
Ossifying fibroma	1	0.1
Osteoid osteoma	5	0.9
Osteomyelitis	34	6.2
Periapical granuloma	12	2.1
Periapical abscess	9	1.6
Pericoronitis	5	0.9
Pleomorphic adenoma	3	0.5
Pyogenic capillary hemangioma	1	0.1
Pyogenic granuloma	14	2.5
Ranula	5	0.9
Radicular cyst	21	3.8
Residual cyst	10	1.8
Squamous cell carcinoma	9	1.6
Squamous papilloma	3	0.5
Traumatic fibroma	33	6
Verrucous hyperplasia	2	0.3
Others	3	0.5

## Data Availability

The datasets used and/or analyzed during the current study are available from the corresponding author.
